# Response to Gelb et al.: “Comparison of tandem mass spectrometry to fluorimetry for newborn screening of LSDs”

**DOI:** 10.1016/j.ymgmr.2017.06.008

**Published:** 2017-07-06

**Authors:** David S. Millington

**Affiliations:** Duke University Health System, Department of Pediatrics, Medical Genetics Division, Durham, NC 27709, USA

Sir:

Gelb et al. [1] have responded to a recent letter [2] refuting claims that tandem mass spectrometry (MS/MS) is superior to digital microfluidic fluorometry (DMF) for newborn screening of LSDs. Their response, however, exemplifies the sources of misinformation that the original letter was intended to highlight [2]. To be explicit, “equivalent cut-offs” is an artificial and unverifiable invention by the authors [3], [4] that has no scientific rationale. Using this argument to manipulate the published data in order to create an alternative conclusion [1], [5] is in my view misleading and deceptive. Furthermore, the previous studies cited [3], [4], [5], [6] are based on retrospective DBS analysis or pilot-phase research. Data reproduced [1] from the comparative Taiwan study [4] purporting to show the superiority of MS/MS over fluorometry are particularly misleading because the methods, reagents and idealized assay conditions (single GAA assays using either solid phase extraction MS/MS or microtiter plate fluorometry, respectively) are far removed from those used for prospective multiple LSD screening in Missouri (DMF) and Illinois (HPLC-MS/MS). Dr. Gelb's letter also states that “there is no correlation seen in one enzymatic activity compared to another… variation in white cell count or other DBS quality factors are not dominant effects” without providing appropriate supporting data. This statement is totally incompatible with multiple peer reviewed publications to the contrary [7], [8], [9], [10]. Newborn screening for LSDs by either MS/MS or DMF results in a relatively high rate of presumptive positive results, because cut-offs are near the low limits of quantification and must be set conservatively to take into account well-documented pre-analytical variables and avoid false negatives [2]. Rational approaches for dealing with this reality include statistical analytical tools as proposed by Rinaldo et al. [11] and/or second-tier tests for DNA analysis and, where possible, other biomarkers [12].
